# Effects of sleep and waking on the synaptic ultrastructure

**DOI:** 10.1098/rstb.2019.0235

**Published:** 2020-04-06

**Authors:** Chiara Cirelli, Giulio Tononi

**Affiliations:** Department of Psychiatry, University of Wisconsin-Madison, 6001 Research Park Blvd, Madison, WI 53719, USA

**Keywords:** cerebral cortex, hippocampus, mouse, serial electron microscopy, spine

## Abstract

We summarize here several studies performed in our laboratory, mainly using serial block-face scanning electron microscopy (SBEM), to assess how sleep, spontaneous waking and short sleep deprivation affect the size and number of synapses in the cerebral cortex and hippocampus. With SBEM, we reconstructed thousands of cortical and hippocampal excitatory, axospinous synapses and compared the distribution of their size after several hours of sleep relative to several hours of waking. Because stronger synapses are on average also bigger, the goal was to test a prediction of the synaptic homeostasis hypothesis, according to which overall synaptic strength increases during waking, owing to ongoing learning, and needs to be renormalized during sleep, to avoid saturation and to benefit memory consolidation and integration. Consistent with this hypothesis, we found that the size of the axon–spine interface (ASI), a morphological measure of synaptic strength, was on average smaller after sleep, but with interesting differences between primary cortex and the CA1 region of the hippocampus. In two-week-old mouse pups, the decline in ASI size after sleep was larger, and affected more cortical synapses, compared with one-month-old adolescent mice, suggesting that synaptic renormalization during sleep may be especially important during early development. This work is still in progress and other brain areas need to be tested after sleep, acute sleep loss and chronic sleep restriction. Still, the current results show that a few hours of sleep or waking lead to significant changes in synaptic morphology that can be linked to changes in synaptic efficacy.

This article is part of the Theo Murphy meeting issue ‘Memory reactivation: replaying events past, present and future’.

## 1. Introduction

In this paper, we discuss the results of several anatomical studies conducted in our laboratory over the last several years, with the goal of characterizing the effects of sleep and waking on synaptic morphology. We start by providing a short overview of the morphological features of excitatory synapses and the evidence showing that there is a strong positive correlation between functional and structural synaptic measures, that is, stronger synapses are also bigger. We then review experiments in mice that assessed changes in spine number after sleep and waking using repeated two-photon imaging. Finally, we focus on recent experiments using serial electron microscopy, which tested the prediction of the synaptic homeostasis hypothesis that synapses should get stronger, and thus bigger, after waking, and weaker, and thus smaller, after sleep. All studies used YFP-H mice whose sleep/waking pattern and response to sleep deprivation were characterized in our laboratory and are also briefly described here.

## 2. Spines and synaptic elements

**Figure 1.**
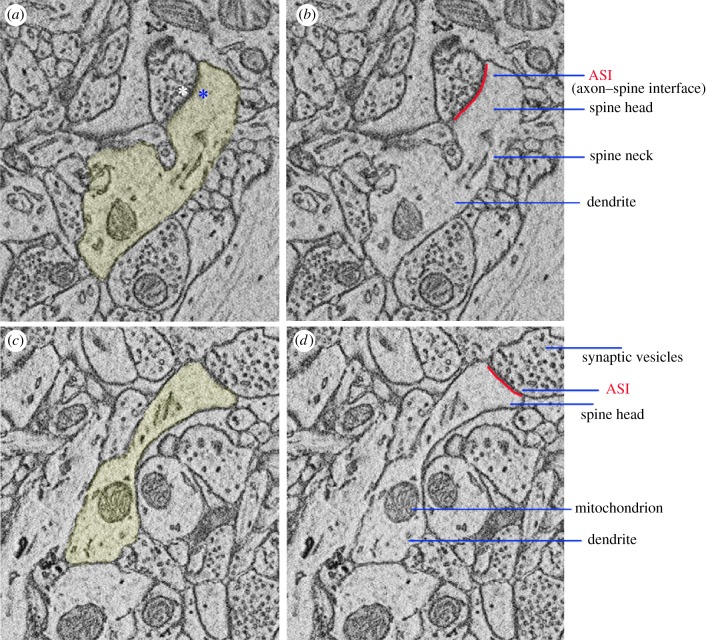
Pre-synaptic and post-synaptic elements. Examples of two spines harbouring synapses in layer 2 of primary motor cortex (mouse, postnatal day 30; [11]). (*a*) and (*c*) show the spines and their corresponding dendritic shafts in yellow. In (*a*), the white asterisk indicates the location of the active zone, facing the post-synaptic density PSD (blue asterisk). (*b*) and (*d*) show other major components of the pre-synaptic and post-synaptic compartments. The direct area of contact between pre-synapse (axon bouton) and post-synapse (spine head) is called the ASI (axon–spine interface), indicated in red.

Most synapses in the mammalian brain are of excitatory, glutamatergic nature and their post-synaptic elements reside in protrusions of the dendritic shafts called spines. Originally named by Cajal, ‘espinas’ were first described in Purkinje cells of the cerebellum and soon afterwards in pyramidal neurons of the cerebral cortex ([1], reviewed in [2,3]). Serial electron microscopy has shown that almost all spines in the adult brain are sites of synaptic contact. In the mouse cerebral cortex, for instance, the complete reconstruction of 144 spines revealed that only 3.6% of them were non-synaptic, that is, they lacked the post-synaptic density (PSD), the electron-dense region where glutamate receptors and scaffold proteins are concentrated [4]. In addition to the PSD, excitatory synapses contain distinct structural elements on the pre-synaptic site, including the synaptic vesicles and the active zone, the specialized area facing the synaptic cleft where vesicles fuse with the pre-synaptic membrane and release glutamate. Ultrastructural studies in the cerebral cortex, cerebellum and hippocampus have found that the number of pre-synaptic vesicles, the size of the active zone, the PSD area, the spine head volume and the area of contact between pre-synapse and post-synapse (also called ASI, axon–spine interface) are strongly positively correlated [5–10]. Some of these pre-synaptic and post-synaptic elements are shown in figure 1.

Structural synaptic measures are also correlated with measures of the strength of a synapse, the efficacy by which a given stimulus is transmitted from the pre-synaptic compartment, the axon of the input neuron, to the post-synaptic compartment, the spine head of the target neuron. Synaptic strength is usually measured by the amplitude of the post-synaptic electric current evoked by that stimulus. Stronger synapses, which yield stronger electric currents triggered by the release of glutamate, also have larger active zones containing more synaptic vesicles, larger PSDs, larger synaptic contact areas between pre- and post-synapse and larger spine heads. For instance, spine head volume and PSD area are correlated with the number of glutamatergic AMPA receptors in the spine [12–14]. Moreover, glutamate uncaging experiments have found that spine head volume is correlated, at the single synapse level, with the amplitude of the electric current mediated by the AMPA receptors present in the spine [15]. Recent experiments using live super-resolution imaging have also revealed that the induction of synaptic potentiation leads to spine expansion and to the coordinated and spatially aligned addition of ‘nanomodules’, clusters of pre-synaptic and post-synaptic proteins that are added to the active zone and the PSD, respectively [16]. Other recent studies combined two-photon uncaging of glutamate, two-photon time-lapse imaging and electron microscopy [10,17]. These experiments found a close correlation between increase in AMPA current and spine expansion after the induction of long-term potentiation in single dendritic spines and described the temporal relation between spine enlargement and the relocation of dozens of synaptic proteins. Spine enlargement was found to occur rapidly after synaptic potentiation and to persist for at least 3 h only in the spines in which the PSD area and the axonal bouton also enlarged, pointing to a close link between the growth of pre-synaptic and post-synaptic components after the induction of synaptic plasticity [10]. In summary, structural and functional measures of synaptic strength are strongly linked, both in baseline conditions and after the induction of synaptic plasticity.

## 3. Characterization of sleep in YFP-H mice

Over the past several years, we have been studying how the number and size of synapses change across the sleep/waking cycle and after sustained sleep loss in B6.Cg-Tg(Thy1-YFP)16Jrs/J mice. In these animals, the yellow fluorescent protein (YFP) is expressed in a subset of cortical dendrites and spines, allowing us to measure spine turnover *in vivo* (see §4 on ‘two-photon imaging’). The sleep/waking pattern of the YFP-H strain was characterized in detail in our laboratory from early adolescence to adulthood (postnatal days P19–111) [18]. In the subsequent ultrastructural studies we mainly focused on one-month-old adolescent animals. At this age, YFP-H mice have consolidated sleep during the day and are mainly awake at night, like adult mice. Their total sleep time and the length of non-rapid eye movement (NREM) sleep episodes reach adult levels at P30, while rapid eye movement (REM) sleep amounts are slightly higher than in adulthood. At the same age, sleep deprivation (4 h starting at light onset) leads to an increase in time spent asleep in the first 4 h of recovery, but there is no significant rebound in slow-wave activity (SWA, 0.5–4 Hz) during NREM sleep. However, as in adults, SWA shows the expected homeostatic decline in the course of baseline NREM sleep. This finding suggests that the lack of SWA rebound after sleep deprivation is due to a ceiling effect, consistent with the very high levels of SWA already present in these animals during baseline [18]. On the other hand, one-month-old YFP-H mice show an increase in theta and alpha frequencies after sleep deprivation, reminiscent of the broad increase in the electroencephalogram (EEG) power spectrum, spanning from 1 to 11 Hz, often seen in adult sleep deprived mice [18]. In summary, sleep/waking pattern and sleep homeostatic regulation in adolescent (P30) YFP-H mice highly resemble those described in adult mice, the most notable difference being the lack of a rebound in SWA after acute sleep loss.

More recently we also characterized sleep/waking behaviour in YFP-H pups aged P13 [19]. EEG patterns are not informative at this age owing to the immaturity of the cortex, and thus sleep and waking were distinguished solely based on behaviour. Two siblings and the dam were observed during the light phase in their cage (sleep/waking behaviour was difficult to assess at night, owing to the small size and dark fur of these mice). YFP-H pups were asleep roughly half of each hour during the day, consistent with the results in a second strain of mice, CD-1, which we also studied at two weeks of age [19]. When forced to stay awake for up to 6 h, YFP-H pups were only weakly responsive to novel objects and gentle handling, in line with the immaturity of hearing and vision at two weeks of age. Yet, during the first 2 h of recovery after sleep deprivation YFP-H pups showed a rebound in sleep duration, also consistent with the results in CD-1 pups. This result strongly suggests that the mechanisms of sleep homeostasis are already in place at two weeks of age, as they are in rats [20,21].

## 4. Two-photon imaging of cortical spine turnover across sleep and waking in YFP-H mice

Several laboratories have applied repeated two-photon imaging to the cerebral cortex of YFP-H mice in order to measure changes in spine turnover caused by sensory experience and learning. These studies found that sensory deprivation induced by whisker trimming preferentially reduces spine elimination in P30 YFP-H mice [22,23], while motor training or sensory enrichment promotes rapid spine formation followed by an increase in both spine formation and elimination [23,24]. We asked whether sleep and waking also affect spine turnover. In adolescent YFP-H animals, aged P23 to P44, we performed repeated two-photon imaging of sensorimotor cortex and followed the formation and elimination of spines in the apical dendrites of layer 5 pyramidal neurons [25]. Based on their shape, spines can be subdivided in three major classes––thin, stubby and mushroom––although this classification is somewhat arbitrary because shapes and sizes of synapses follow a continuum [8,26]. We found that spine formation and elimination occurred in small- and medium-sized spines (thin and stubby), but not in large (mushroom) spines. Both processes happened at all times, independent of behavioural state, but sleep and waking biased spine turnover. Specifically, spine elimination exceeded spine formation when 6–8 h of sleep occurred between the two imaging sessions. By contrast, spine gain was greater than spine loss when mice spent most of the intervening time spontaneously awake at night, or when they were sleep deprived with novel objects during the day. Filopodia, thin protrusions without a bulbous head that are more frequent in immature brains, were present in all mice but showed no consistent changes due to sleep and waking. Spine number also showed no net changes when only 2–3 h of sleep or waking occurred between two consecutive imaging sessions. Thus, spine formation can be triggered rapidly by learning [27] but, at least in naive animals, several hours of sleep seem to be required to promote net spine loss. This interpretation is supported by independent observations performed in another laboratory, which found that spine elimination was higher across the 12 h light period, when mice are mostly asleep, than over the dark period, when they are mainly awake [28].

Along a dendritic segment, synaptic strength depends both on the total number of synapses and on their size. The two-photon imaging experiments performed in YFP-H mice only assessed changes in spine number. Thus, they cannot be used in isolation to infer changes in synaptic strength, for two reasons. First, owing to the suboptimal spatial resolution of the imaging method, spine size was not measured in these experiments. Second, acute changes in spine number do not necessarily reflect changes in synapse number, because the majority of newly formed spines either do not form synapses or represent transient synapses that disappear within 2–4 days [29]. For instance, one study in naive adult mice found that 71% of the spines formed within the previous 24 h lacked detectable amounts of PSD-95, the major scaffold protein of the PSD that anchors glutamate receptors at the synapse, and only 18% of these spines were still present 24 h later [30]. Moreover, in trained animals, the consolidation of the newly learned task does not correlate with the acute increase in spine turnover but with the stabilization of a subset of newly formed synapses, which requires a few days and coincides with the acquisition of a cluster of PSD-95 that stabilizes AMPA receptors (reviewed in [29]).

## 5. Three-dimensional electron microscopy of cortical and hippocampal spines in YFP-H mice

**Figure 2.**
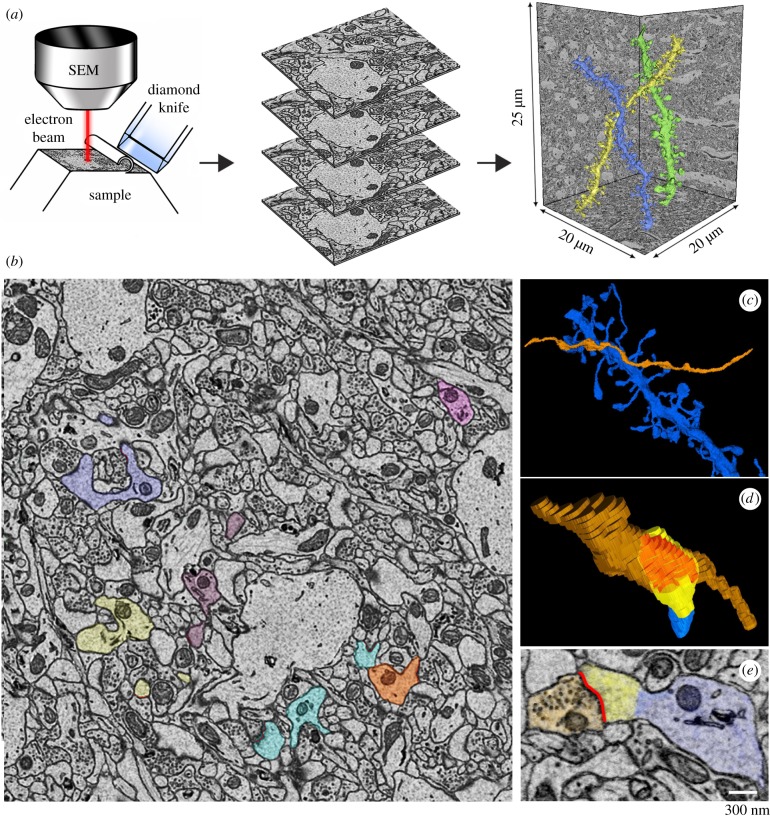
(*a*) Schematic showing the basic principle of serial block-face scanning electron microcopy: the face of the sample block is imaged and sliced repeatedly. Consecutive images are collected in stacks that are automatically aligned and then used for the manual segmentation of the structures of interest; for instance, the three dendritic branches shown in colour on the right (CA1 stratum radiatum, mouse P30; [32]). SEM, scanning electron microscope. (*b*) Example of one two-dimensional image in the stack where several dendritic elements and spines were manually segmented (CA1 stratum radiatum, mouse P30; [32]. Each dendrite and its spines are indicated by a different colour. Trained annotators learned to use the open source software Fiji to manually trace the object of interest (spine, ASI, axon bouton, etc.) over serial sections, to obtain a three-dimensional reconstruction of the object. All final reconstructions were checked for accuracy and consistency by the same person, to ensure that all annotators followed the same established rules. (*c,d*) Three-dimensional representation of a dendritic segment (in blue) and the axon making synaptic contact (orange); the lower panel shows one spine head (yellow) contacted by the axon (orange); their direct area of contact, the ASI, is indicated in red. (*e*) Two-dimensional image showing an example of a segmented axon bouton with synaptic vesicles (orange), ASI (red), spine head (yellow) and dendritic segment (blue).

In order to accurately assess synapse size after sleep and waking, we applied serial block-face scanning electron microscopy [31]. One cannot follow the same synapses longitudinally with this method, but its exquisite spatial resolution allows measurement of both synapse number and size with great accuracy. The microscope is equipped with an automated microtome that cuts thin sections (usually 40–50 nm thick) from the surface of a block of tissue previously fixed and stained with heavy metals. After the face of the block is scanned, a section is cut and the block is raised to the focal plane and imaged again, allowing the automatic acquisition of hundreds of serial images (figure 2*a*). We routinely acquire stacks of∼500 images (approx. 10 000–15 000 µm^3^) in less than 24 h. The stacks are then segmented by trained annotators to reconstruct the dendritic shafts, the spines and their organelles, and the ASI (figure 2*b–e*). In some of our studies, the synaptic vesicles and the peripheral astrocytic processes surrounding the synapses were also reconstructed and measured.

Because the accuracy of the automatic methods of segmentation remains suboptimal, we perform all three-dimensional reconstructions manually. This time-consuming step is currently the major factor that limits the number and size of the brain regions to be analysed. In the past 6 years, we have completed three studies that are discussed below performed in the cerebral cortex [11] and the CA1 region of the hippocampus [32] of one-month-old adolescent YFP-H mice, and in the cerebral cortex of two-week-old YFP-H pups [19]. In these experiments, the size of synaptic components was measured after several hours of sleep, spontaneous waking and enforced waking. Overall, the results show that many cortical and hippocampal synapses get smaller after sleep compared to waking, consistent with an overall decrease in synaptic strength after sleep [33]. Other studies have further analysed a subset of these synapses to document changes in the surrounding peripheral astrocytic processes [34,35] as well as measured the effects of sleep loss on myelin formation [36].

## 6. Changes in the ultrastructure of cortical synapses in adolescent YFP-H mice after sleep and waking

In the first study, we reconstructed a total of 7149 cortical spines containing excitatory synapses from 3 groups of one-month-old YFP-H mice [11]. The brains were collected in the light phase after approximately 7 h of sleep (S) or extended waking enforced by exposure to novel objects (EW), and after approximately 7 h of spontaneous waking at night (SW). The use of three experimental groups allowed us to tease apart the effects of behavioural state (sleep versus waking) from those of stress (spontaneous versus forced waking) and time of day (day versus night). We targeted layer 2 in primary motor (M1) and primary sensory (S1) cortex, because superficial layers of primary areas remain highly plastic even after early development (e.g. [37–41]). We segmented a total of 168 spiny dendritic branches, which in layer 2 belong to basal and oblique dendrites of layer 2 pyramidal neurons, or to intermediate and terminal dendrites of layers 3 and 5 pyramidal neurons. Synapse size was first assessed by measuring the ASI, which was fully reconstructed in 6305 synapses (≥277 ASIs/mouse, 4 mice/group). Because sleep/waking effects in M1 did not differ from those in S1, the results were pooled.

The main finding was that ASI size declined on average by approximately 18% after sleep relative to after both waking conditions, which were indistinguishable from each other. At the population level, the decline in ASI size could be described as downscaling, that is, synapses shrank after sleep in a manner proportional to their size (multiplicative scaling). The downscaling was widespread but selective: it did not affect all synapses but a range of sizes comprised between 0 and 80%, sparing the largest 20% of synapses. In addition to small/medium size, the presence of endosomes inside the spine was another predictor of downscaling after sleep, in line with the role of these organelles in protein recycling and secretory trafficking inside the post-synaptic compartment [42,43]. By contrast, downscaling could not be predicted by the presence of a spine apparatus in the spine, or mitochondria in the axonal bouton. Consistent with the ASI results, the spine head volume also decreased after sleep relative to both waking conditions. The density of cortical synapses (number of synapses by dendrite surface area) did not change between sleep and waking, nor did the density of the cortical spines lacking a synapse (filopodia). In summary, in layer 2 of M1 and S1 all but the largest excitatory synapses declined in size after sleep compared to waking.

## 7. Changes in the ultrastructure of hippocampal synapses in adolescent YFP-H mice after sleep and waking

The three experimental groups (S, EW and SW) employed in the study of cortical synapses were then used to test whether sleep and waking also affect hippocampal synapses [32]. We targeted the middle of the stratum radiatum of the CA1 region, the focus of many previous studies on synaptic plasticity. In this area, axospinous synapses are established by Schaffer collaterals from ipsilateral CA3 pyramidal neurons, by collaterals from contralateral CA3 pyramidal neurons, and by associational fibres from CA3 cells in other lamellae [44]. In total, we reconstructed 7819 spines containing a synapse in 101 dendritic branches, and the ASI was fully traced and measured in 7341 synapses (≥425 ASIs/mouse, 4–6 mice/group).

**Figure 3.**
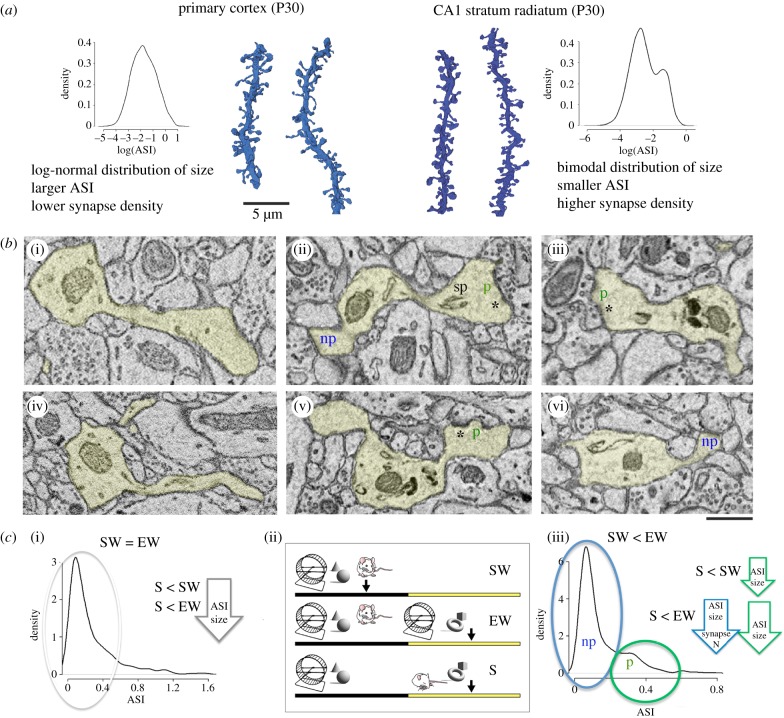
(*a*) Examples of dendritic segments from primary cortex (left, lighter blue, S group) and CA1 (right, darker blue, S group) and lists of some of their structural differences. Probability densities (log-transformed) reveal a log-normal distribution of ASI size in cortex and a bimodal distribution in CA1. Results are described in detail in the original publications: primary cortex P30 [11]; CA1 stratum radiatum P30 [32]. (*b*) Two-dimensional images of cortical synapses ((i) and (iv)) and CA1 synapses ((ii), (v) and (iii),(vi)). (ii), (v) and (iii),(vi) show the two types of CA1 synapses, non-perforated (np, in blue) and perforated (p, in green), and some of the distinctive features of perforated synapses, including the presence of a spine apparatus (sp) and a discontinuous post-synaptic density (asterisk). Non-perforated synapses have a continuous post-synaptic density. (*c*) Probability density of ASI size (μm^2^) in cortex (left) and in CA1 (right) and summary of the results. In primary cortex, the decrease in ASI size after sleep is size-dependent: it occurs in small and medium synapses (grey circle and arrow), which represent approximately 80% of synapses, but spares the largest synapses. In CA1, the perforated synapses (green circle and arrows) show smaller ASI size after sleep (S) relative to spontaneous waking (SW) as well as after S relative to extended waking (EW). Non-perforated synapses (blue circle and arrow) show smaller ASI size after S relative to EW (but not relative to SW). Synapse density shows a trend to decrease in S relative to EW (*p* = 0.0832; details in [32].) The three experimental conditions are depicted in the (ii) (SW, EW and S) and are the same in the two studies.

In M1 and S1, we found that the distribution of synapse size was log-normal, that is, most synapses were small or medium size and only a few synapses were large, in line with the findings reported in other cortical areas [30,45,46]. The distribution of synapse size in the CA1 region was instead bimodal: it included a more numerous group of small synapses and a less numerous, but sizable, group of medium and large synapses (figure 3*a*). The two groups partially overlapped in size, but could be distinguished using specific ultrastructural features, in line with previous studies [47,48]. Specifically, the more numerous group included the ‘non-perforated’ synapses, which are on average small, have a continuous PSD, lack a spine apparatus, and are weak because they contain few AMPA and NMDA receptors. The less numerous group included the ‘perforated’ synapses, which are on average large, located in mushroom spines, have discontinuities in their PSD (hence the name perforated), often contain a spine apparatus and/or a small protrusion called spinula, and are strong because they house many AMPA and NMDA receptors (figure 3*b*).

The effects of sleep and waking on these two types of synapses were complex. Perforated synapses did not change in number between sleep and waking, and their ASI was on average larger after spontaneous waking relative to sleep, and even larger after forced waking relative to sleep. In these synapses, the differences in ASI size between sleep and waking could be explained by scaling, at least at the population level, as in cortex. On the other hand, neither the density nor the ASI size of non-perforated synapses changed between sleep and spontaneous waking but they both increased after sleep deprivation relative to the other two conditions. In the non-perforated synapses, the differences in ASI size between sleep and waking could not be explained by scaling.

The synaptic effects of sleep and waking in cortex and CA1 are summarized in figure 3*c*. Overall, CA1 synapses were smaller after sleep, consistent with the results in cortical synapses, but unlike in cortex, the effects of sleep and waking in the hippocampus varied based on synapse type and on waking condition. By contrast to the cortex, the difference in ASI size between sleep and spontaneous waking was restricted to the perforated synapses, which on average are bigger and likely account for most of the overall synaptic strength [49]. This may seem surprising since the largest 20% of cortical synapses did not change between sleep and waking, presumably because they were more stable and, as suggested in other studies, already committed to previous memories (e.g. [50,51]). However, we found that CA1 synapses are smaller than cortical synapses, consistent with previous studies, and thus even the largest CA1 synapses may still be far from saturation. Another difference between cortex and hippocampus was that the changes in synapse size in CA1 were, in the case of non-perforated synapses, accompanied by changes in synapse number. One recent study estimated that at least half of synapses in primary cortex are permanent, while most if not all CA1 synapses in the adult mouse brain have a lifetime of only one to two weeks [52]. In another recent study, the spine turnover of CA1 synapses was even higher, approximately 40% within 4 days [53]. Thus, CA1 synapses may be more dynamic than cortical synapses, consistent with the role of the hippocampus in novelty detection [54,55] and with the transient nature of hippocampal-dependent memories [56].

ASI changes in CA1 could be explained by scaling only in the case of perforated synapses, whereas in cortex downscaling after sleep applied to all the synapses that showed differences between sleep and waking (80%). Because the presence or absence of scaling in our data can only be tested at the population level, these differences are difficult to interpret. New imaging methods that can detect longitudinal changes in synapse strength *in vivo* may be informative [57]. Finally, relative to sleep the changes in CA1 synapses were much more pronounced after sleep deprivation than after spontaneous waking, and the two waking conditions also differed from each other, unlike in cortex. By design, EW mice were encouraged to explore all the time and had little quiet waking, contrary to SW mice. CA1 neurons can respond in a graded manner to different levels of novelty [54,55] and therefore may have been engaged more during sleep deprivation, which was enforced by exposure to novel objects, than during spontaneous waking. Moreover, sharp waves/ripples are more frequent during stereotyped behaviours such as grooming, eating and drinking than during exploration [58,59]. Thus, they likely occurred more frequently during spontaneous, non-exploratory waking than during sleep deprivation, in which active exploration was continuously induced by the presentation of novel objects. Sharp waves/ripples have been involved in mediating synaptic downscaling during sleep [60] and may have contributed to synaptic renormalization in SW mice. However, there is no direct evidence supporting this interpretation, and the synaptic consequences of sharp waves/ripples are likely to vary between waking and sleep [61,62], given the different neuromodulatory milieu in the two behavioural states. Indeed, the higher levels of noradrenalin, dopamine, acetylcholine, orexin and serotonin during waking relative to sleep are a key factor that explains why waking promotes learning and synaptic potentiation, as discussed in previous reviews [33,63]. Higher levels of glucocorticoids in waking relative to sleep may also promote optimal performance and behavioural adaptation and, when increased at moderate levels such as during the physiological wake state, may contribute to the net increase in synaptic strength observed in this behavioural state [33,63].

## 8. Changes in the ultrastructure of M1 synapses in YFP-H pups after sleep and waking

**Figure 4.**
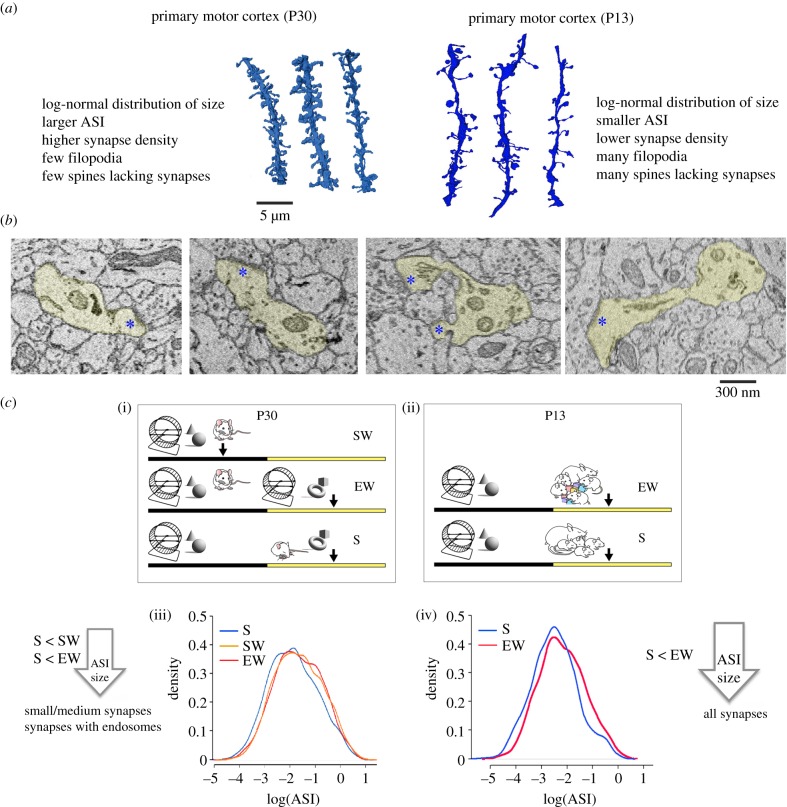
(*a*) Examples of dendritic segments from primary motor cortex (M1) in P30 mice (left, lighter blue, S group) and in P13 pups (right, darker blue, S group) and lists of some of their structural differences. Results are described in detail in the original publications: primary motor cortex P30 [11]; primary motor cortex P13 [19]. (*b*) 2D images of M1 synapses in P13 pups (layer 2). Blue asterisks indicate the post-synaptic density. (*c*) Summary of the results in M1 cortex in P30 mice (left) and P13 pups (right). (i) and (ii) show the experimental groups used in the two studies: SW, spontaneous waking; EW, extended waking; S, sleep. The SW group is missing at P13 because pups at this age are never spontaneously awake for several hours. (iii,iv) Probability density of ASI size (log-transformed) at P30 (left) and P13 (right), plotted separately for each experimental group to show the shift to left of the S group. At P30, the decrease in ASI size after S relative to both SW and EW occurs in small and medium synapses and synapses that contain endosomes, but spares the largest synapses. At P13, the decrease in ASI size after S relative to EW occurs in all synapses, independent of their size or the presence of endosomes.

The decline in synapse size associated with sleep was observed at one month of age, after most developmental changes in cortex and hippocampus had already occurred. It was not known, however, whether sleep would lead to a similar decrease in synapse size during early development, when the wiring of cortical circuits still occurs at significant pace and spine turnover is high. To address this question, we used YFP-H pups, aged P13 [19]. Their brain is still undergoing significant developmental changes and sleep and waking can be distinguished reliably using behavioural criteria. We compared cortical spines in layer 2 of primary motor cortex after 5–6 h of sleep and after 5–6 h of sleep deprivation (five mice/group). Unlike in the previous studies, we could not include an SW group, because pups at this age are never spontaneously awake for several hours. We fully reconstructed 3750 spines in M1 (136 dendritic segments), and in 2506 of them, the ASI was measured. As expected, synapse density and ASI size were on average smaller in pups than in adolescent mice. Moreover, at P13, a significant proportion of spines did not form a synapse (27%), in contrast with approximately 12% at P30 (figure 4*a,b*).

The synaptic effects of sleep and waking at P13 closely resembled those at P30. As in adolescent mice, spine density and synapse density in pups did not differ between sleep and extended waking, although a trend was present for more spines lacking synapses after sleep. The main finding was that ASI size declined after sleep compared with after sleep deprivation. Moreover, at the population level, the decline in ASI size could be described as downscaling. However, the average decline in ASI after sleep was larger at P13 than at P30 (approx. 34% versus approx. 18%). Furthermore, in pups, we found no evidence for size-dependent scaling: all synapses, including the largest ones, decreased in size after sleep. Because even the largest synapses at P13 were smaller than those at P30, this result suggests that synapses are not fully committed at two weeks of age, not even the largest synapses, and thus may keep changing in size across the sleep/waking cycle. In summary, the decrease in ASI size after sleep was larger at P13 than at P30 and occurred in more synapses (figure 4*c*). At P13, the brain is still growing in size and one possibility was that such an immature brain may not need synaptic renormalization as much as the mature brain, in which the total number of synaptic connections is stable and there is little, if any, space to grow. The opposite scenario, however, was that the need to maintain the balance in overall synaptic strength was especially high during early development, when many synapses are still forming or growing, and especially in response to the rich waking experience of our pups, which were exposed to new objects for the first time in their life [33,63]. Our results are more in line with the second possibility and suggest that, at least in the supragranular layers of primary motor cortex, sleep-dependent renormalization is needed during the periods of enhanced brain plasticity during development. This conclusion fits with the main tenet of the synaptic homeostasis hypothesis, according to which sleep is ‘the price for plasticity’: the greater the net increase in synaptic strength due to learning, the higher is the need to rebalance overall synaptic strength during sleep. If so, one would predict that specific synapses, such as the thalamocortical synapses targeting layer 4 of primary cortices, should show strong sleep/waking modulation in their strength until the end of the critical period but less so afterwards, when their ability to undergo plastic changes is decreased and can be reinstated only by specific manipulations [39,64–67].

## 9. Conclusion

The changes in the synaptic ultrastructure during sleep and waking do not happen in isolation. For example, we found that the peripheral astrocytic processes surrounding cortical axospinous synapses also undergo modifications [34]. In layer 2 of primary motor cortex, more than 80% of spines were contacted by peripheral astrocytic processes and in most of them (approx. 75%) the contact reached the ASI, that is, was close to the synaptic cleft. Large, mushroom spines were more likely to be contacted by an astrocytic process, and the extent of the astrocytic coverage increased with spine size, likely because large spines harbour larger and stronger synapses, which in turn are more in need of astrocytic support. Relative to sleep, the proportion of spines with astrocytic processes touching the ASI increased after both spontaneous and extended waking. This result likely reflects the decreased neuronal activity in the cerebral cortex during sleep compared with waking, leading to the reduced need for ionic balance and glutamate clearance. This finding also suggests that although both neurons and astrocytes undergo structural changes across the sleep/waking cycle, these changes affect overlapping but not identical populations of spines: changes in synapse size occur in small and medium synapses but spare the largest ones, whereas the largest spines are the most likely to show changes in their astrocytic processes.

Together, the experiments summarized above were performed as a stringent test of the synaptic homeostasis hypothesis, according to which overall synaptic strength is poised to increase during waking, owing to ongoing learning, and needs to be rebalanced during sleep when the brain is disconnected from the environment [33]. Given the strong positive correlation between structural and functional measures of synaptic strength, this hypothesis predicts that synapses should be on average smaller after sleep. This is indeed what we found, with interesting differences between cortical and hippocampal synapses at P30. Of note, these differences suggest that global factors such as the higher brain temperature during waking as compared with sleep are unlikely to mediate the synaptic changes observed in cortex and CA1 in adolescent mice. If this was the case, the changes would be expected to occur always in all synapses and to be always more pronounced after forced waking than after spontaneous waking, since sleep deprivation increases brain temperature above baseline levels [68], but this is not what we found.

A major future challenge will be to extend these observations to other brain regions, to adult and old brains, and to determine the shortest period of sleep required for these structural changes to occur. In all the studies described above, we compared several hours of waking with several hours of total sleep, without distinguishing between NREM sleep and REM sleep, because our goal was to assess the net, overall effect of consolidated sleep. Ultrastructural changes are slow, and we assume that the changes that we observed are the net result of several NREM/REM cycles. It is unlikely that a single episode of NREM sleep, or of REM sleep, which in mice lasts only a few minutes, will have a net effect on the ultrastructure, although it may be enough to affect the expression of AMPA receptors on the surface of the spine head. In fact, an important question for the future will be to understand how sleep and waking affect synapses and, as a consequence, memory, cumulatively, over several days, and whether such cumulative effects may be critical for the integration of new with old memories.

## Data accessibility

This article does not contain any additional data.
